# Multi-psychotropic drug prescription and the association to neuropsychiatric symptoms in three Norwegian nursing home cohorts between 2004 and 2011

**DOI:** 10.1186/s12877-016-0287-1

**Published:** 2016-06-01

**Authors:** Christine Gulla, Geir Selbaek, Elisabeth Flo, Reidun Kjome, Øyvind Kirkevold, Bettina S. Husebo

**Affiliations:** Centre for Elderly and Nursing Home Medicine, Department of Global Public Health and Primary Care, University of Bergen, PO Box 7804, N-5018 Bergen, Norway; Centre for Old Age Psychiatric Research, Innlandet Hospital Trust, PO Box 2136, N-3103 Tonsberg, Ottestad Norway; Norwegian National Advisory Unit on Ageing and Health, Vestfold Hospital Trust, Tonsberg, Norway; Faculty of Medicine, Institute of Health and Society, University of Oslo, Oslo, Norway; Research group in Social Pharmacy, Department of Global Public Health and Primary Care, and Centre for Pharmacy, University of Bergen, Bergen, Norway; Faculty of Health, Care and Nursing, Gjovik University College, Gjovik, Norway; Municipality of Bergen, Bergen, Norway; Department of Global Public Health and Primary Care, University of Bergen, PO Box 7804, N-5018 Bergen, Norway; Faculty of Health, Care and Nursing, Norwegian University of Science and Technology (NTNU) in Gjøvik, N-2821 Gjøvik, Norway

**Keywords:** Dementia, Nursing homes, Neuropsychiatric symptoms, Psychotropic drugs, Antidepressants, Antipsychotics, Hypnotics, Anxiolytics, Anti-dementia drugs

## Abstract

**Background:**

Neuropsychiatric symptoms, such as affective symptoms, psychosis, agitation, and apathy are common among nursing home patients with and without dementia. Treatment with one or more psychotropic drug is often without explicit clinical indication, despite low treatment efficacy, and potential side effects. We aim to investigate the multi-psychotropic drug use to identify factors and patient characteristics associated with multi-use.

**Methods:**

We analysed three cohorts from 129 Norwegian nursing homes, collected between 2004 and 2011. Patients (*N* = 4739) were assessed with the Neuropsychiatric Inventory – Nursing Home version (NPI-NH), Clinical Dementia Rating scale, and Physical Self Maintenance Scale. We used ordinal logistic regression to analyse associations between psychotropics (antidepressants, antipsychotics, anxiolytics, hypnotics, and anti-dementia drugs), patient characteristics, and neuropsychiatric symptoms.

**Results:**

Patients used on average 6.6 drugs; 27 % used no psychotropics, 32 % one, and 41 % multiple psychotropic drugs (24 % two, 17 % ≥3). Thirty-nine percent were prescribed antidepressants, 30 % sedatives, 24 % anxiolytics, and 20 % antipsychotics. The total NPI-NH score was associated with multi-use (OR 1.02, 95 % CI 1.02–1.03), and increased from a mean of 13.5 (SD 16.3) for patients using none, to 25.5 (21.8) for patients using ≥3 psychotropics. Affective symptoms (depression and anxiety) were most strongly associated with multi-psychotropic drug use (OR 1.10, 95 % CI: 1.09–1.12). Female gender, independency in daily living, younger age, dementia, and many regular drugs were also associated with multi-use.

**Conclusion:**

Forty-one percent were exposed to multi-psychotropic drug prescriptions. Contrary to current evidence and guidelines, there is an extensive use of multiple psychotropic drugs in patients with severe NPS and dementia.

## Background

Neuropsychiatric symptoms (NPS), such as agitation, psychosis, depression, anxiety, and sleep problems, are frequently observed among nursing home (NH) patients with and without dementia [1, 2]. Approximately 90 % of all people with dementia experience NPS during the course of the disease [2]. These symptoms are distressing for the patient, the relatives, and the caregivers [3].

People with dementia and NPS are often treated with psychotropic drugs [4], despite national and international warnings concerning severe side effects [5, 6]. Recent prevalence rates reflected widespread use; for example, Nijk et al. investigated psychotropic drug use in 1322 NH patients from 59 dementia care units in the Netherlands [7]. Psychotropic drugs were used by 63 % of the patients, 20 % used two, and 7 % used three or more. Antipsychotics were the most prevalent of the psychotropics, used by 37 % of the patients. Another cross-sectional study in Belgian NHs showed that 79 % received psychotropic drugs, of which 54 % were benzodiazepines, and 33 % antipsychotics [8]. Our own Norwegian trend analysis of six cross-sectional NH cohorts reported a considerable increase in the use of psychotropics, from 58 % in 1997 to 71 %, in 2009 [9]. Antidepressants increased most (from 32 to 51 %), followed by anxiolytics and hypnotics (from 15 to 23 % and 22 %, respectively). The only drugs not increasing in use were typical antipsychotics and benzodiazepine hypnotics. The use of two different psychotropics increased from 17 to 22 % and three different drugs from 5 to 11 %.

Psychotropic drugs are often prescribed without proper symptom assessment, without any clear indications, and treatment persists for longer than recommended [10]. In addition, review articles investigating the benefit of psychotropic drugs on NPS found minor to no treatment effects [4, 11, 12]. Two reviews on the discontinuation of psychotropics in older adults demonstrated that discontinuation decreased the incidence of falls and delirium, and the NPS remained stable [13, 14]. A randomized placebo controlled trial on discontinuation of antidepressants in nursing home patients found that 86 % could discontinue successfully, however there was increased depressive symptoms in the intervention group compared to the controls [15]. Another randomized placebo controlled trial on the effect of sertraline in major depressive episodes in patients with Alzheimer disease, found that sertraline was superior to placebo in treating major depressive disorders [16]. These studies indicate an effect of antidepressants given the right indication is present, and the importance of weighting potential benefit to harm.

Treatment safety is another important factor because increased mortality rates were found for people with dementia using atypical antipsychotics [17]. The antipsychotic withdrawal trial (DART-AD) on Alzheimer’s patients from NHs in the UK even showed that survival for the discontinuation group was not only higher after 12 months, but the difference in survival increased with time [18]. Increased mortality, and adverse events such as falls, stroke, and fractures have also been related to antidepressants; serious side effects were even more prevalent for selective serotonin reuptake inhibitors compared to tricyclic antidepressants [19]. Another population-based study (*N* = 906 422) showed an increased risk of hip-fractures in users of hypnotics and anxiolytics [20].

Despite the considerable focus on psychotropic use in general, little attention has been given to multi-psychotropic drug use and its association with the severity of NPS. This is of key importance because the combination of several psychotropics does not necessarily ameliorate the symptoms, and may increase the risk of interactions and side-effects. In the present study, we investigated patient characteristics to find associations to the use of multiple-psychotropic drugs. We hypothesized that severe NPS and its sub-syndromes are associated with increased psychotropic drug use. We expected the factors age, sex, dementia, and independency in activities of daily living to be associated with the use of more than one psychotropic.

## Method

### Subjects

We included patients, 65 years and older, from three Norwegian cross-sectional multi-centre NH studies from 2004, 2007, and 2011. Norwegian NHs are managed by the municipalities and are an essential part of the primary health care system. All patients in the included wards were eligible if they had lived in the NH for more than 2 weeks prior to data collection and consented to participate. Demographics, diagnoses and information on prescribed medicines were collected from the patients’ medical records. Standardized interviews of nurses, who knew the patients well, were used to collect data on clinical status.

The 2004 cohort included patients in 26 NHs from 18 municipalities in four counties, 1165 patients were eligible, two declined to participate, and 26 were too young, giving a total of 1137 patients. Data was originally collected to explore the association between NPS and psychotropic drug use at different stages of dementia [21]. Before data collection, registered nurses were trained during a 2-day programme on structured interviews for primary caregivers.

The 2007 cohort examined patients living in 63 NHs in the South-Eastern health region of Norway. In all, 151 wards were included, with 2108 eligible patients, 162 had a stay less than 2 weeks, 3 declined to participate, and 64 were too young, giving 1879 patients in total. This study investigated the problematic practice of administering medication to patients by concealing them in food and beverages [22]. Registered nurses received 2 h of training in addition to oral and written instructions on how to complete data collection. This study did not collect data on diagnoses.

The 2011 cohort consisted of 23 of the 26 NHs, which were included in the 2004 cohort, along with 40 new NHs from 31 municipalities altogether. For patients assessed in both 2004 and 2011, we used only data from 2004. Altogether, 2385 patients were eligible, 98 were also assessed in 2004, 423 declined to participate, 17 died before assessment, 33 were terminally ill or had a serious somatic condition, one moved, 53 were excluded without giving a cause, and 37 were too young, giving 1723 patients. The 2004 procedure for inclusion, education and collection of data was used [23].

### Outcome measures

The Neuropsychiatric Inventory – NH version (NPI –NH) is a 12-item proxy rated instrument to assess the frequency and severity of NPS over the last 4 weeks in people with and without dementia [24]. NPI-NH consists of items for delusions, hallucination, agitation, depression, anxiety, euphoria, apathy, disinhibition, irritability, aberrant motor behaviour, night-time behaviour, and eating disturbances. Items are scored on frequency (0 to 4 – absent to daily) and severity for the patient (1 to 3 – mild to severe). Severity and frequency are multiplied to create a sum-score from 0 to 12 for each item. The Norwegian translation has good validity and reliability [25]. In this study, items were clustered into the clinical relevant sub-syndromes, following a previous exploratory factor analysis, psychosis (hallucination and delusions), affective symptoms (depression and anxiety), and agitation (agitation and irritability). Apathy did not cluster with any of the sub-syndromes [26].

The Clinical Dementia Rating Scale (CDR) assesses the level of dementia [27, 28]. The CDR score is calculated with an algorithm giving extra weight to memory problems. CDR scores of 0, 0.5, 1, 2, and 3, indicate no, possible, mild, moderate, and severe dementia, respectively. In the 2007 cohort CDR 0.5 was scored as 0.

The patients’ dependency in daily living was measured with the Physical Self-Maintenance Scale (PSMS). The six items toileting, feeding, dressing, grooming, physical ambulation, and bathing yield an aggregate score of 6–30; higher scores indicate higher dependency in activities of daily living. Good reliability and validity of PSMS has been reported earlier [29, 30].

### Medication and diagnoses

The psychotropic drugs were classified as antipsychotics (N05A), anxiolytics (N05B), sedatives (N05C), antidepressants (N06A), and anti-dementia drugs (N06D) in the Anatomical Therapeutic Chemical Index (ATC) classes [31]. “Regular drugs” was a count of the drugs the patient was prescribed on the day of data collection, pro re nata drugs were not included in this count. “Regular drugs without psychotropics” was the number of regular drugs, when psychotropic drugs were subtracted. The diagnoses were coded with International Classification of Diseases – 10th version [32]. Dementia is an aggregate of the diagnoses F00 dementia with Alzheimer, F01 vascular dementia, F02 dementia in other diseases classified elsewhere, and F03 unspecified dementia.

### Statistical methods

Background characteristics and comparisons between the cohorts were analysed using chi-square for categorical variables, and one-way ANOVA for continuous variables. The total NPI-NH score was log-transformed in the ANOVA due to non-normality. Association between CDR and dementia was tested using chi square test and Phi for strength of association. The patients were divided into four ordinal groups according to number of psychotropic prescriptions (0, 1, 2 and ≥3). We used cumulative odds ordinal logistic regression with proportional odds analyses [33] to create odds ratios (OR), 95 % confidence intervals (CI), and *p*-values for the association between the prescription of psychotropic drugs and age, gender, severity of dementia, NPS, dependency in activities of daily living, regular drugs without psychotropics, and diagnoses. We created two logistic regression analyses (Analysis 1 and 2) because the 2007 cohort did not contain information on diagnoses and NPS-NH total score and the sub-syndromes can cause a potential multicollinearity problem. Both regression analyses included age, gender, regular drugs without psychotropics, dependency, and cohort. Analysis 1 also contained severity of dementia measured by CDR and the sub-syndromes of NPS (affective symptoms, agitation, psychosis, and apathy). Analysis 2 covered the NPI-NH total score and the most frequent diagnoses. We adjusted for cohort year in the analyses due to differences in the demographics between the cohorts; the cohort was included as a nominal independent variable (2011 as reference). Both analyses met the assumption of proportional odds and there was no multicollinearity. Statistical significance was set to *p* < 0.05. IBM SPSS statistics version 22.0 (IBM Corp, Armonk, NY, USA) was used to perform the analyses.

## Results

In total, 4739 participants were included from three cohorts of 129 Norwegian NHs, assessed in 2004, 2007, and 2011. Demographics and clinical characteristics stratified by cohort-year are shown in Table 1. The mean age was 86 years; 71 % of the patients were women, and they used on average 6.6 drugs. Age (*p* = 0.03) and regular drugs (*p* < 0.001) differed significantly between the cohorts, with higher age and more drugs in 2011. Dementia (46 %), hypertension (23 %), stroke (16 %), heart failure (13 %), atrial fibrillation (13 %), osteoporosis (9 %), hip fracture (9 %), angina (9 %), and diabetes (8 %) were the most frequent diagnoses in the medical records. Assessed by CDR, 80 % of the patients had dementia (CDR ≥1). There was a small (Phi = 0.274), but statistically significant (*p* < 0.001) association between dementia according to CDR and a diagnosis of dementia in the patient records. CDR identified 1307 (98 %) of the patients with a diagnosis of dementia in the records (*N* = 1328). Of the patients with no recorded diagnose of dementia (*N* = 3411), 2547 (75 %) were classified with dementia according to CDR. The mean (SD) PSMS and NPI-NH scores were 18.1 (5.4) and 17.5 (18.7), respectively. Dementia, dependency and NPI-NH total score differed between the cohorts (*p* < 0.05), with more dementia and lower dependency in 2011, and lower NPI-NH score in 2007 (Table 1).

**Table 1:** Demographics of the study population in total and by cohort

	Total	2004	2007	2011	*P*-value
	*N* = 4739	*N* = 1137	*N* = 1879	*N* = 1723	
Age, mean (SD) ^a,b^	85.7 (7.0)	85.0 (6.5)	85.9 (7.1)	85.9 (7.1)	0.03
Women, N (%)	3383 (71)	836 (74)	1321 (70)	1226 (71)	NS
Regular drugs without psychotropics, mean (SD)^a,^ ^b,c^	5.2 (2.9)	4.7 (2.8)	5.1 (2.9)	5.7 (3.0)	<0.001
Dementia (CDR), N (%)					<0.001
No	497 (11)	37 (3)	406 (22)	54 (3)	
Questionable	388 (8)	183 (16)	0 (0)	205 (12)	
Mild	686 (15)	225 (20)	163 (9)	298 (18)	
Moderate	1336 (28)	306 (27)	529 (28)	501 (30)	
Severe	1786 (38)	382 (34)	768 (41)	636 (38)	
Dependency, mean (SD)^b,d^	18.1 (5.4)	18.0 (5.3)	18.5 (5.5)	17.7 (5.3)	<0.001
NPI-NH total score, mean (SD)	17.5 (18.7)	18.5 (19.2)	16.1 (17.6)	18.5 (17.6)	0.03

ANOVA for continuous and *X* ^2^ for categorical data

*NPI-NH* neuropsychiatric inventory – nursing home version, *SD* standard deviation, *NS* not significant

*CDR* Clinical Dementia Rating scale 1 to 3, ^a^significant between 2004 and 2007, ^b^significant between 2004 and 2011, ^c^significant between 2007 and 2011, ^d^higher scores indicates more dependency in daily living

### Psychotropic drug use

Almost three out of four received at least one psychotropic drug and two out of five received two or more (range 0–7) (Table 2). Thirty-nine percent received antidepressants, 30 % sedatives, 24 % anxiolytics, 20 % antipsychotics, and 14 % anti-dementia drugs. The most frequent psychotropic drug combinations were antidepressants and sedatives (14 %), antidepressants and anxiolytics (12 %), and sedatives and anxiolytics (11 %). The most prescribed individual drug was the z-hypnotic, zopiclone (23 %). This medication was combined with oxazepam in 6 % of the patients, and with escitalopram in 4 % (Table 3).

**Table 2:** Clinical characteristics associated with psychotropic drug use, crude and adjusted values for Analysis 1 and 2

	Psychotropic drugs				Crude		Adjusted	
	0	1	2	≥3	OR	95 % CI	OR	95 % CI
Patients, %	27.3	32.2	23.4	17.1				
*Factors included in Analyses 1 and 2*								
Age, mean (SD)	86.7 (6.9)	86.1 (7.0)	85.3 (6.8)	83.9 (6.8)	0.97^**^	0.96–97	0.97^**^	0.96–0.98
Females^a^, %	68.8	72.4	72.5	72.8	1.13^*^	1.01–1.27	1.20^*^	1.06–1.36
Dependency, mean (SD) ^d^	18.9 (5.5)	18.1 (5.5)	17.8 (5.2)	17.2 (5.2)	0.97^**^	0.96–0.98	0.96^**^	0.95–0.97
Regular drugs without psychotropics, mean (SD)	4.9 (2.8)	5.3 (3.0)	5.5 (2.9)	5.2 (2.9)	1.04^**^	1.02–1.06	1.05^**^	1.03–1.07
*Analysis 1*								
Cognitive impairment^b^								
No^a^, %	9.9	11.1	11.0	10.3	1		1	
Questionable, %	9.2	7.7	8.2	8.0	0.88	0.70–1.12	1.19	0.91–1.55
Mild, %	13.3	15.6	14.1	15.7	1.03	0.84–1.27	1.23	0.98–1.54
Moderate, %	26.0	28.6	31.1	28.5	1.05	0.87–1.26	1.19	0.97–1.46
Severe, %	41.6	37.1	35.7	37.6	0.89	0.74–1.06	1.15	0.92–1.42
NPI-NH sub-syndromes								
Affective symptoms, mean (SD)	1.8 (3.5)	2.6 (4.2)	3.9 (5.2)	5.9 (6.2)	1.^**^	1.10–1.13	1.10^**^	1.09–1.12
Agitation, mean (SD)	3.2 (5.2)	3.5 (5.5)	4.4 (6.1)	5.7 (6.5)	1.05^**^	1.04–1.06	1.02^**^	1.01–1.03
Psychosis, mean (SD)	1.8 (4.1)	2.3 (4.6)	2.8 (5.0)	4.4 (6.3)	1.06^**^	1.05–1.07	1.02^**^	1.01–1.04
Apathy, mean (SD)	1.9 (3.5)	1.8 (3.2)	2.0 (3.4)	2.3 (3.6)	1.02^*^	1.00–1.03	0.99	0.98–1.01
*Analysis 2, 2004 and 2011 cohort*								
NPI-NH total score	13.5 (16.3)	15.4 (16.8)	19.2 (19.3)	25.5 (21.8)	1.02^**^	1.02–1.02	1.02^**^	1.02–1.03
Diagnoses^c^								
Dementia, %	27.4	26.9	26.5	33.3	1.13^*^	1.01–1.27	1.19^*^	1.03–1.37
Hypertension, %	14.4	13.5	14.2	12.4	0.93	0.81–1.08	1.01	0.86–1.19
Stroke, %	12.2	9.3	9.2	7.3	0.72^**^	0.61–0.86	0.89	0.74–1.07
Heart failure, %	9.3	7.8	7.3	7.0	0.82^*^	0.68–0.99	0.90	0.73–1.11
Atrial fibrillation/flutter, %	8.5	8.3	6.8	5.7	0.78^*^	0.64–0.94	0.92	0.75–1.13
Angina, %	3.9	4.7	6.1	6.1	1.42^*^	1.13–1.79	1.60^**^	1.26–2.04
Osteoporosis, %	5.7	6.0	4.7	4.8	0.85	0.68–1.08	0.91	0.72–1.16
Hip fracture, %	6.1	5.0	4.8	5.3	0.88	0.70–1.10	1.04	0.82–1.33
Diabetes, %	5.8	5.2	5.4	2.8	0.75^*^	0.59–0.95	0.75^*^	0.59–0.97

*P*-values, odds ratios and confidence intervals from cumulative odds logistic regression. Analysis 1 and 2 included age, sex, dependency and total medication. The factor unique to each analysis is listed below their headlines in the table

*NPI-NH* neuropsychiatric inventory – nursing home version, *SD* standard deviation, *OR* odds ratio, *CI* confidence interval

^a^used as reference, ^b^assessed by Clinical Dementia Rating scale, ^c^from ICD-codes in patient records, ^*^ *p*-value < 0.05, ^**^ *p*-value < 0.001, ^d^higher scores indicates more dependency in daily living

**Table 3:** Use of psychotropic drug groups, individual psychotropics, and combinations of psychotropics

ATC code and generic name	*N* = 4739, %
N05A Antipsychotics	19.9
N05A X08 Risperidone	5.6
N05A D01 Haloperidol	3.4
N05A H03 Olanzapine	2.8
N05B Anxiolytics	23.9
N05B A04 Oxazepam	19.2
N05B B01 Hydroxyzine	3.2
N05B A01 Diazepam	2.1
N05C Sedatives	30.5
N05C F01 Zopiclone	23.0
N05C D02 Nitrazepam	3.4
N05C M02 Clomethiazole	2.7
N06A Antidepressants	39.2
N06A B10 Escitalopram	12.5
N06A B04 Citalopram	10.7
N06A X03 Mianserin	7.4
N06D Anti-dementia drugs	13.7
N06D A02 Donepezil	6.8
N06D X01 Memantine	4.7
N06D A03 Rivastigmine	2.0
Combinations of psychotropic drug classes	
Antidepressants and sedatives	14.4
Antidepressants and anxiolytics	12.1
Sedatives and anxiolytics	10.6
Antidepressants and antipsychotics	9.1
Two antidepressants	6.2
Combination of specific psychotropic drugs	
Zopiclone and Oxazepam	5.6
Zopiclone and Escitalopram	3.5
Zopiclone and Citalopram	3.3
Oxazepam and Escitalopram	3.0
Oxazepam and Citalopram	2.8
Oxazepam and Mianserin	2.5
Zopiclone and Mianserin	2.2

### Factors associated with multi-psychotropic drug use

**Fig. 1:**
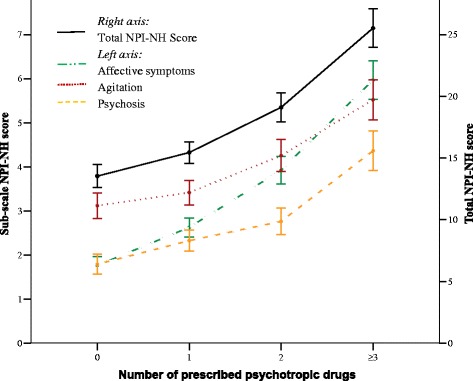
Difference in neuropsychiatric symptoms for patients using 0, 1, 2 or ≥3 psychotropics. Mean NPI-NH scores (95 % CI) for the sub-syndromes affective symptoms, agitation and psychosis (left- axis, *dotted colored lines*), and mean total NPI-NH score (right axis, *solid black line*). X-axis represents patients using 0 to ≥3 psychotropic drugs. *NPI-NH* Neuropsychiatric Inventory – Nursing Home version, *CI* confidence interval

**Fig. 2:**
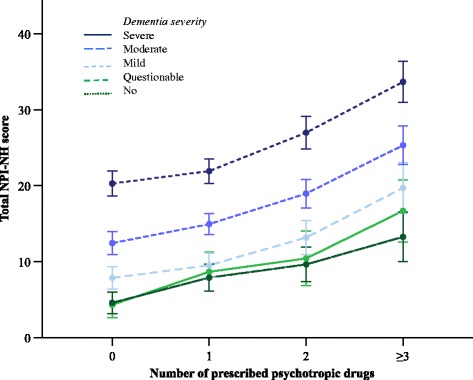
Neuropsychiatric symptoms and cognitive function for patients using 0, 1, 2 or ≥3 psychotropics. Mean total NPI-NH score scores for different levels of cognitive function measured with CDR. X-axis represents patients using 0 to ≥3 psychotropic drugs. *NPI-NH* Neuropsychiatric Inventory – Nursing Home version, *CI* confidence interval, *CDR* Clinical Dementia Rating Scale

The total NPI-NH score was associated with multi-use (OR 1.02, 95 % CI 1.02–1.03), and increased from a mean of 13.5 (SD 16.3) for patients using none, to 25.5 (SD 21.8) for patients using ≥3 psychotropics (Table 2). The sub-syndromes affective symptoms (OR 1.10, 95 % CI 1.09–1.12), agitation (OR 1.02, 95 % CI 1.01–1.03), and psychosis (OR 1.02, 95 % CI 1.01–1.04) demonstrated a significant association as well (Fig. 1). The adjusted OR for variables included in both Analysis 1 and 2 were similar and had the same effects; the OR listed here are from Analysis 1. Female gender (OR 1.20, 95 % CI 1.06–1.36), independency in daily activities (OR 0.96, 95 % CI 0.95–0.97), younger age (OR 0.97, 95 % CI 0.96–0.98), and more regular drugs without psychotropics (OR 1.05, 95 % CI 1.03–1.07) were all associated with increased psychotropic drug use. Compared to no diagnosis of dementia in their records (Analysis 2), having dementia increased the likelihood of being treated with multiple psychotropics (OR 1.19, 95 % CI 1.03–1.37) (Table 2). Poorer cognitive function measured with CDR was not associated with the use of psychotropics compared to normal cognitive function (Fig. 2). Of the other frequent diagnoses, diabetes lead to less psychotropics (OR 0.75, 95 % CI 0.59–0.97), while angina lead to more (OR 1.60, 95 % CI 1.26–2.04) (Table 2). The 2004 and 2007 had significantly higher risk of multi-use than the 2011 cohort, with the highest OR for the 2007 cohort (OR 1.65, 95 % CI 1.45–1.89).

## Discussion

The present study found that 41 % of Norwegian NH patients were treated with two or more psychotropic drugs. Antidepressants (39 %) were most prevalent and often combined with sedatives (14 %) and anxiolytics (12 %). In accordance with our hypotheses, patients with severe NPS, especially depression and anxiety, used more psychotropics. Female gender, independency in daily activities, younger age, dementia, and more regular drugs, were all associated with multi-use.

High prevalence of multiple psychotropic drug use was also demonstrated by other NH studies. In a Dutch study, 27 % used two or more psychotropic drugs, and antipsychotics were the most prescribed drugs (37 %) [7]. An American study found that 45 % used two or more psychotropics, and antidepressants were most frequently used (41 %) [34]. A study from Sweden found that 28 % used ≥3 psychotropics, and 51 % used hypnotics [35].

The association between psychotropic drug use and NPS has been highlighted earlier [7, 36, 37]. This was supported by our study; we found that severe NPS, especially affective symptoms and agitation, were related to multi-use. The use of multiple psychotropic drugs might indicate that clinicians try to treat a symptom with one drug, and if the symptom persists, they add another drug, often with the same indication, without stopping or re-evaluating the treatment. This can be due to belief in the additive effect, or to the burden NPS puts on patients and caregivers, resulting in a pressure from caregivers to prescribe [37, 38]. The OR for the NPI items were low, for instance for affective symptoms it was 1.10. This reflects an increase of 10 % in odds of receiving more psychotropics for every unit change on the NPI-NH score. This implies that patients with severe symptoms received considerably more psychotropics.

The current study found that especially patients with depression and anxiety used more psychotropics. Indication for the drugs and appropriateness of their use is beyond the scope of this article. However, both depression and anxiety are prevalent among NH residents, and drugs for these conditions are commonly prescribed [39]. A recent study showed that the antidepressants were prescribed for other indications than depression in 50 % of the cases, and most patients lacked a diagnostic work-up [40]. Affective symptoms may have many causes, hence the assessment and treatment should be adjusted accordingly to avoid over-prescribing of psychotropics [41]. We also found independency in daily living to be associated with the use of more psychotropics. A possible reason for this could be that patients with high dependency are often bedridden or so reduced that they are incapable of causing the same level of commotion and staff distress as the mobile and active independent patients. Hence, the independent patients receive more psychotropics to dampen their symptoms, which might explain this association. Other studies show divergent associations [34, 36, 42].

Our results showed that people with a diagnosis of dementia in their records use more psychotropics than those without a diagnosis, despite the consensus that they should be treated with utmost caution and less psychotropic drugs [41, 43, 44]. An article describing the 2004 cohort found that only 55 % of patients with dementia according to the CDR had a dementia diagnosis in the medical records in the nursing home [21]. The weak association between a dementia diagnosis and psychotropics might be an effect of staff blaming the dementia for the NPS, rather than investigation other causes for these symptoms [12]. Patients with a diagnosis of diabetes used fewer psychotropic drugs. This corroborates and extends earlier research that people with a high Charlson’s Comorbidity Score received fewer antipsychotics [45].

### Limitations and strengths

There are some limitations to this study. Indications, duration, or doses were not recorded, nor were pro re nata medications. These factors, and the cross-sectional design, made it impossible to assess the appropriateness and effects of the drugs. We did not know whether the patient used the drugs because of severe NPS, if the NPS was reduced by the drugs, or the drugs aggravated NPS. Another limitation is that the diagnoses listed in the medical records may be of low quality because of the absence of a standard of what to report and of sub-optimal electronic patient records [46]. The use of popular, validated and reliable assessment scales for NPS, dementia and ADL [24, 25, 27–30], are a major strength and make our results comparable to other studies. Age, percentage of women, drug use, and CDR scores were similar to other Norwegian nursing home studies [40, 47, 48]. The large cohort size, including patients from 129 NHs all over the country, from small and large municipalities, thus very likely mirrors the Norwegian NH population.

## Conclusion

Forty-one percent of NH patients use multiple psychotropic drugs. Severe NPS, especially affective symptoms, were associated with extensive use. The use of multiple psychotropic drugs in in patients with dementia is widespread, contrary to current evidence and guidelines. There is an urgent need to test and implement methods to optimize prescription procedures for these patients.

## Abbreviations

ATC, anatomical therapeutic chemical index; CDR, clinical dementia rating scale; CI, confidence intervals; ICD-10, international classification of diseases 10th version; NDPA, the Norwegian data protection authority; NH, nursing home; NPI-NH, the neuropsychiatric inventory – nursing home version; NPS, neuropsychiatric symptoms; OR, odds ratio; PSMS, physical self maintenance scale; REC-SE/E, the regional committees for medical and health research ethics: South-East and East; SD, standard deviation; SHDir, the directorate for health and social affairs.
